# Mutations I117V and I117M and Oseltamivir Sensitivity of Pandemic (H1N1) 2009 Viruses

**DOI:** 10.3201/eid1801.111079

**Published:** 2012-01

**Authors:** Aeron C. Hurt, Sook Kwan Leang, David J. Speers, Ian G. Barr, Sebastian Maurer-Stroh

**Affiliations:** World Health Organization Collaborating Centre for Reference and Research on Influenza, North Melbourne, Victoria, Australia (A.C. Hurt, S.K. Leang, I.G. Barr);; PathWest Laboratory Medicine, Nedlands, Western Australia, Australia (D.J. Speers);; Agency for Science, Technology and Research, Singapore (S. Maurer-Stroh);; Ministry of Health, Singapore (S. Maurer-Stroh);; Nanyang Technological University, Singapore (S. Maurer-Stroh)

**Keywords:** oseltamivir, drug resistance, zanamivir, I117V, I117M, peramivir, H275Y, synergistic, mutations, NAI, neuraminidase inhibitor, influenza, viruses

## Abstract

Analysis of mutations I117V and I117M in the neuraminidase of influenza A pandemic (H1N1) 2009 viruses showed that I117V confers a mild reduction in oseltamivir sensitivity and has a synergistic effect of further increasing resistance when combined with H275Y. Contrary to recent reports, the I117M mutation does not alter oseltamivir sensitivity.

The neuraminidase inhibitors (NAIs) oseltamivir and zanamivir have been widely used in many countries to treat infection with influenza A pandemic (H1N1) 2009 virus. Since the start of the pandemic, >27,000 strains have been tested for oseltamivir resistance, of which 383 (1.4%) have contained the H275Y neuraminidase (NA) mutation, the same mutation that was found in oseltamivir-resistant prepandemic seasonal subtype H1N1 viruses that emerged in 2007–08. Apart from the H275Y mutation, a small number of other NAI resistance mutations have been detected in the NA of pandemic (H1N1) 2009 viruses, such as S247N (*1*), I223V (*2*), and I223R (*3*). Another NA mutation, I117M, has also recently been associated with oseltamivir resistance in pandemic (H1N1) 2009 viruses (*4**,**5*). The I117M NA mutation was detected in a virus from South Korea that was isolated from a patient before oseltamivir treatment and then was detected, with the H275Y mutation, in the same patient after oseltamivir treatment, although the dual mutations were not conclusively shown in the same virus (*4**,**5*). In both studies, neither the I117M variant nor the dual I117M + H275Y strain were specifically tested for oseltamivir resistance. Instead, the viruses were assumed to be resistant on the basis of previous studies that described a reduction in oseltamivir sensitivity in influenza A (H5N1) viruses because of an I117V amino acid substitution (*6**,**7*), rather than the I117M substitution detected in their studies.

Other studies have documented that different amino acid substitutions at key NA residues can cause considerably different effects on NAI susceptibility and that these mutations can have a variable effect in different NA backgrounds (*8*). Therefore, we tested the assumption that the I117M mutation confers oseltamivir resistance and investigate the role of the I117M and the I117V mutations with and without the H275Y mutation in pandemic (H1N1) 2009 viruses.

## The Study

By using site-directed mutagenesis and reverse genetics as described (*9*), we generated recombinant viruses with NA from the pandemic (H1N1) 2009 virus A/Auckland/1/2009 and the remaining genes from A/PR/8/34. Recombinant viruses were constructed with no NA mutations, with single I117V or I117M NA mutations, and with dual I117V + H275Y or I117M + H275Y NA mutations.

NAI sensitivity analysis with a fluorescence-based NA inhibition assay (*10*) found that compared with a recombinant with no mutations, the I117V mutation conferred a 5-fold increase in the oseltamivir concentration required to inhibit 50% (IC_50_) of the NA activity, a 2-fold increase in zanamivir IC_50_, and no change in peramivir IC_50_. In comparison, the I117M mutation had no effect on sensitivity to any of the NAI drugs (Table 1).

**Table 1:** NAI sensitivity of naturally occurring pandemic (H1N1) 2009 virus I117V mutant and I117V, I117M, I117V + H275Y, I117M + H275Y, and H275Y reverse genetics variants*

Pandemic (H1N1) 2009 viruses	Mutation	Zanamivir			Oseltamivir carboxylate			Peramivir	
		Mean IC_50_ ± SD, nmol/L	-fold difference†		Mean IC_50_ ± SD, nmol/L	-fold difference†		Mean IC_50_ ± SD, nmol/L	-fold difference†
Mean of NAI-sensitive viruses‡	–	0.28 ± 0.15	–		0.45 ± 0.35	–		0.20 ± 0.10§	–
A/Perth/504/2010	I117V	0.96 ± 0.28	3		1.63 ± 0.70	4		0.09 ± 0.01	1
RG-WT	–	0.24 ± 0.05	–		0.30 ± 0.20	–		0.09 ± 0.01	–
RG-I117V	I117V	0.54 ± 0.12	2		1.42 ± 0.52	5		0.08 ± 0.02	1
RG-I117M	I117M	0.32 ± 0.05	1		0.31 ± 0.06	1		0.11 ± 0.01	1
RG-I117V + H275Y	I117V + H275Y	0.44 ± 0.05	2		568.84 ± 54.89	1,896		47.08 ± 32.57	523
RG-I117M + H275Y	I117M + H275Y	0.29 ± 0.04	1		163.72 ± 17.76	546		16.12 ± 0.84	179
RG-H275Y	H275Y	0.26 ± 0.03	1		195.02 ± 21.05	650		19.72 ± 1.42	219

*NAI, neuraminidase inhibitor; IC_50_, 50% inhibitory concentration; –, not applicable. †-fold differences compared with IC_50_ value of RG-WT, except for A/Perth/504/2010, which was calculated for on the basis of comparison to the mean IC_50_ of circulating pandemic (H1N1) 2009 viruses. ‡n = 3,334, obtained through World Health Organization Global Influenza Surveillance and Response System, April 2009–June 2011. §Mean and SD of peramivir IC_50_ values were based on analysis of 273 isolates. RG strains were derived by using site-directed mutagenesis and reverse genetics. Mean IC_50_ ± SD values of the A/Perth/504/2010 virus and the RG strains were calculated on the basis of values derived from 3 independent assays.

The dual I117V + H275Y variant had oseltamivir and peramivir IC_50_ values that were 3× and 2× higher, respectively, than the IC_50_ of a virus with the H275Y mutation alone. In contrast, the IC_50_ of the I117M + H275Y variant was not substantially different from that of the H275Y mutant for all of the NAIs, further demonstrating the lack of effect of the I117M mutation on NAI sensitivity.

Analysis of 3,334 pandemic (H1N1) 2009 strains received at the World Health Organization (WHO) Collaborating Centre, Melbourne, Victoria, Australia, through the WHO Global Influenza Surveillance and Response System from April 2009 through June 2011, showed that 1 isolate had a I117V NA mutation, but no I117M variants were detected. The I117V variant, A/Perth/504/2010 (GenBank accession nos. HA:EPI279165 and NA:279164; www.gisaid.com), was isolated from a 5-year-old boy and had a 4-fold and 3-fold reduction in sensitivity to oseltamivir and zanamivir, respectively, similar to that of the RG-I117V strain (Table 1). Neither the patient nor his family members or siblings were undergoing any NAI treatment.

Apart from the A/Perth/504/2010 strain, no other pandemic (H1N1) 2009 strains with an I117V NA mutation were reported on GenBank or public sequence databases, demonstrating the high degree of conservation at this residue. However, 45 NA sequences from highly pathogenic influenza A (H5N1) strains in the public sequence databases contained the I117V mutation. The I117V mutation in highly pathogenic influenza A (H5N1) viruses has previously been reported to confer a 5- to 16-fold reduction in oseltamivir sensitivity and up to a 4-fold reduction in zanamivir sensitivity (*6**,**7*).

Residue I117 is not in direct structural contact with oseltamivir, although it has neighboring residues that are known to affect drug susceptibility such as E119, R118, and V116. By using the predictive computational force field FoldX (*11*) in YASARA (*12*), we modeled the effects on structural stability of I117V, I117M, H275Y, I117V + H275Y, and I117M + H275Y mutations in the pandemic (H1N1) 2009 NA crystal structure (Protein Data Bank no. 3NSS; www.pdb.org) (*13*). The model estimated local destabilization effects for the known oseltamivir-resistance mutation H275Y, which served as a control for the approach, whereas substantially smaller effects for I117V were observed, and almost no stability change was predicted for the I117M mutation (Table 2). The estimated local destabilization effects for the dual mutations H275Y + I117M and H275Y + I117V were not substantially different from that predicted for the H275Y mutation alone. When the NA inhibition assay IC_50_ data (Table 1) were compared with the estimated local destabilization effects of the mutants (Table 2), a good correlation was demonstrated between the 2 methods, although functional testing showed a larger difference between the H275Y and the H275Y + I117V variants than that estimated in the computational model.

**Table 2:** Predicted local structure destabilization for the different NA mutations from pandemic (H1N1) 2009 viruses*

NA mutation	Mean ± SD level of destabilization, kcal/mol†
H275Y	4.7 ± 0.4
H275Y + I117V	5.2 ± 0.4
H275Y + I117M	5.0 ± 0.3
I117V	0.5 ± 0.02
I117M	0.1 ± 0.1

*NA, neuraminidase. †Calculated with FoldX (*11*) on the basis of 5 repetitions.

**Figure 1:**
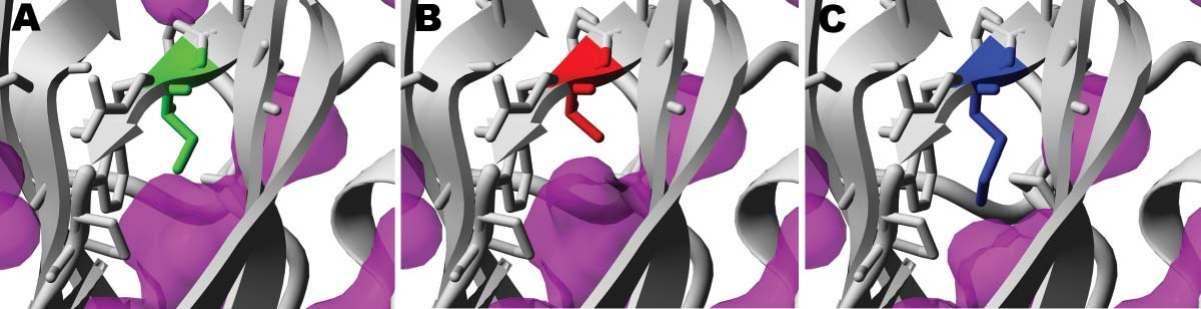
Structural details of neuraminidase mutations from pandemic (H1N1) 2009 viruses. A) Wildtype mutation I117 (green). B) mutation I117V (red). C) I117M (blue). All were modeled with FoldX (*11*) in YASARA (*12*) in the context of the pandemic (H1N1) 2009 virus neuraminidase crystal structure (Protein Data Bank: 3nss). Side chains of residues <3 Å of residue 117 are shown as sticks. Cavities within the structure (1.4 Å radius water probe) are shown in magenta.

**Figure 2:**
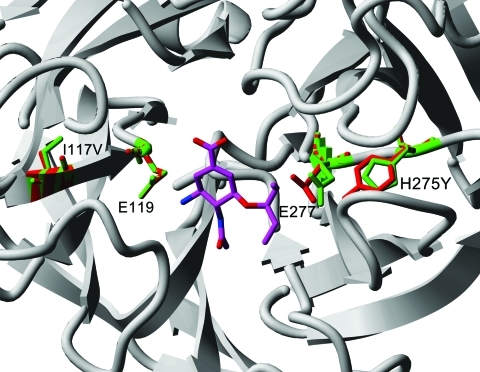
Comparison of wildtype I117 mutation from pandemic (H1N1) 2009 viruses (green residues; Protein Data Bank: 3nss) with FoldX/YASARA model of I117V + H275Y double mutant (red residues) (*11*,*12*). Residue numbering is according to pandemic (H1N1) 2009 neuraminadase. Oseltamivir is added as reference (Protein Data Bank: 3clO) and shown in magenta.

The destabilization effect of I117V appears to be mainly caused by the increase in an internal cavity (Figure 1), which could increase flexibility of neighboring residues that form part of the drug-binding framework. The H275Y and I117V mutations are at opposite sides of the binding pocket (Figure 2) and, although they are not expected to affect each other’s side-chain environment directly, the simultaneous changes on both sides of the drug show more effects on oseltamivir binding than the single mutations alone.

## Conclusions

Although the I117V mutation was detected in 1 isolate from Australia, analysis of sequences from public databases shows that it is extremely rare in pandemic (H1N1) 2009 viruses to date. Although the I117V mutation causes a mild reduction in oseltamivir sensitivity, on the basis of pharmacokinetic data, we expect that a variant carrying this mutation would not be clinically resistant (*14**,**15*). However, in combination with H275Y, the I117V mutation has a synergistic effect on oseltamivir resistance, raising the oseltamivir IC_50_ to 3× that caused by the H275Y mutation alone and to a level that is likely to be clinically important. Previous studies have reported that the I117M mutation may confer oseltamivir resistance (*4**,**5*), although in this study we have demonstrated that this is not the case. These results therefore highlight the importance of assaying functional drug resistance when reporting novel mutations because resistance cannot be assumed on the basis of data from other amino acid substitutions at the same residue.
